# Optimal Sensor Arrangements in Angle of Arrival (AoA) and Range Based Localization with Linear Sensor Arrays

**DOI:** 10.3390/s130912277

**Published:** 2013-09-12

**Authors:** Sanvidha C. K. Herath, Pubudu N. Pathirana

**Affiliations:** Networked Sensing and Control Laboratory, School of Engineering, Deakin University, Pigdons Road, Geelong, Victoria 3217, Australia; E-Mail: pubudu@deakin.edu.au

**Keywords:** Angle-of-Arrival, range, linear array

## Abstract

This paper investigates the linear separation requirements for Angle-of-Arrival (AoA) and range sensors, in order to achieve the optimal performance in estimating the position of a target from multiple and typically noisy sensor measurements. We analyse the sensor-target geometry in terms of the Cramer–Rao inequality and the corresponding Fisher information matrix, in order to characterize localization performance with respect to the linear spatial distribution of sensors. Here in this paper, we consider both fixed and adjustable linear sensor arrays.

## 1. Introduction

Different techniques can be used to localize an emitting or non-emitting target [1–4]. AoA and range-based localizations are common passive measurement techniques where the location of an emitter is obtained by triangulation of bearing or range information collected at a number of sensors. These techniques have numerous potential applications in radar, unmanned aerial vehicles (UAVs) and mobile positioning in wireless telecommunication systems.

The Stansfield estimator in [5] is considered as one of the first localization methods. It is a weighted least-squares (LS) estimator that assumes small independent Gaussian distributed bearing noise with no sensor location error. The pseudolinear estimator (PLE) introduced in [6] relaxed the prior knowledge requirement of the emitter range by the Stansfield estimator. For Gaussian distributed bearing noise, the passive emitter localization problem can be converted into a nonlinear LS estimation problem by engaging the maximum likelihood approach. In [7] the nonlinear LS problem was linearized by Taylor series expansion resulting an iterative Gauss–Newton algorithm.

The potential performance of any particular localization algorithm is highly dependent on the relative sensor-target geometry [6,8]. This performance is determined by the geometry of the physical aperture or array as well as the weighting that can be applied to measurements [9]. A partial characterization of the sensor-target geometry with various matrices related to Cramer–Rao inequality or the corresponding Fisher information matrix has been explored in [10]. Since the Cramer–Rao lower bound is a function of the relative sensor-target geometry, a number of authors have attempted to identify underlying geometrical configurations that minimize some measure of this variance lower bound in [6,10–17]. In [12] the problem of moving the sensors in order to track moving targets while maintaining an optimal localization geometry is examined. Indeed, for mobile sensor-based localization problems, a similar measure of localization performance can be used to identify optimal sensor trajectories and to derive control laws for navigating sensors along such trajectories [2,6,12,14,15,18–20]. Localization of targets via AoA sensors are currently being studied in many robotic applications [21,22]. A modulo conversion method to finding phase ambiguity and consequently estimating the AoA of an electromagnetic wave incident on a multi-element interferometer with non-uniform spacings is presented in [23]. Furthermore, some array design formulas are given in [24].

Determining the optimal trajectory for a single moving platform with an AoA sensor was explored in [25]. In this approach, the optimal trajectory was determined by maximizing the determinant of the Fisher Information Matrix (FIM), which minimizes the uncertainty of the overall estimation. In this work it is assumed that the estimation algorithm is nearly efficient, because its error covariance matrix is close to the Cramer–Rao Lower Bound (CRLB) [26]. Deriving and dealing with actual Mean Squared Error (MSE) expressions for AoA/range localization methods can be challenging due to the nonlinear nature of the estimation problem. Hence FIM can be used to simplify the analysis to a greater extent [11,13].

Most of the existing literature concerned on the placement of AoA/range sensors around the target for optimal localization [11,13], but linear sensor arrays play a crucial role in some real world applications [8,27–38]. A new array geometry, which is capable of significantly increasing the degrees of freedom of linear arrays, is introduced in [39].

In this paper we provide a more rigorous characterization of the relative sensor-target geometry for linear sensor arrays based on AoA-only and range-only localization, and to the best of our knowledge, no such analysis exists in the literature.

In our approach we consider the localization problem involving a single target and multiple adjustable AoA/range sensors located as a linear array (uniform and non-uniform). In this case, the Cramer–Rao lower bound with the corresponding Fisher information determinant is used to investigate the optimality of the relative sensor-target geometry, exploring the intrinsic relation with the spatial diversity and the underlying measurement model.

The remainder of this paper is organized as follows. In Section 2, an outline of some notations and conventions together with the problem formulation for AoA-only and range-only localization is given. Section 3 provides results for optimality of the localization for linear sensor arrays that utilise the AoA sensor measurements, while Section 4 gives the results for the range sensor measurements. Section 5 provides simulation results for our theoretical derivations and the paper concludes with a discussion of the optimal configurations in Section 6. In fact, the results presented in this paper provide fundamental information about how the localization performance is affected by the sensor-target geometry for linear sensor arrays. This information is of significant value to users of multiple sensor (linear arrays) based localization systems.

## 2. Problem Formulation and Assumptions

**Figure 1.**
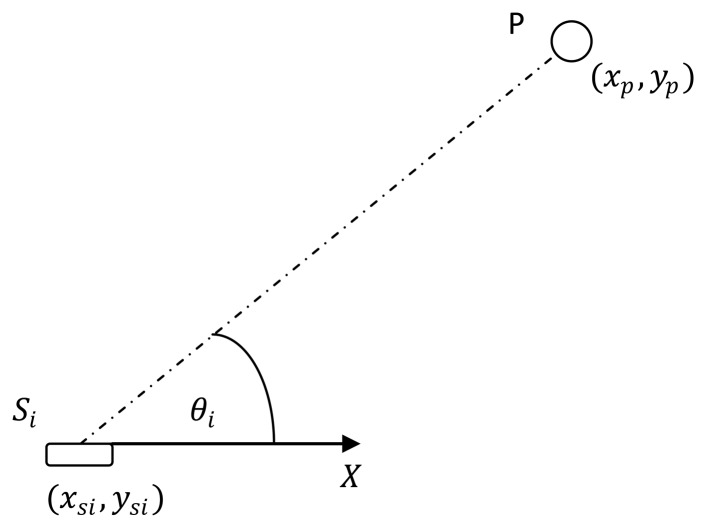
Measurement from a sensor.

Consider the *i^th^* sensor of a multiple AoA/range sensors located in a linear array that are positioned to localize a single stationary target (Figure 1). The unknown location of the target is given by *p* = [*x_p_y_p_*]*^T^*. The AoA/range sensors are marked as *i* ∈ {1,2,…, *N*} and *N* ≥ 2 with the location of the *i^th^* sensor given by *S_i_* = [*x_si_y_si_*]*^T^*. The distance between the sensor *S_i_* and the target *P* is given by *r_i_* = *‖p* — *S_i_‖*. The bearing *θ_i_* from sensor *S_i_* to the target is measured clockwise form *x*-axis such that *θ_i_*(*p*) ∈ [0, 2π).

In general, the set of measurements from *N* sensors can be written as *ẑ* = *z*(*p*) + *n*, where *z*(*p*) = [*z_1_*(*p*) *… z_n_*(*p*)]*^t^* and *n* = [*n*_1_*… n_N_*]*^T^*. It is assumed that the measurement errors of distinct sensors are independent of each other. For simplicity, it is assumed that the error variances of multiple distinct sensors are equal and given by $\sigma_{z}^{2}$. The covariance matrix for *n* sensors is then given by $R_{z}=\sigma_{z}^{2}I_{N}$, where *I_N_* is an N-dimensional identity matrix. The general measurement vector *ẑ* can thus be considered as an *observable* normally distributed random *vector* and can be described by *ẑ* ∼ 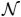
(*z*(*p*), *R_z_*).

The Cramer–Rao inequality lower bounds the covariance achievable by an unbiased estimator under two mild regularity conditions. Considering the unbiased estimate *p̂* for *p*, the Cramer–Rao bound states that

(1) $$E[(\hat{p}-p){\left(\hat{p}-p\right)}^{T}]\geq {ℐ}^{-1}(p)=C(p)$$

where ℐ is the Fisher information matrix. In general, if ℐ is singular then no unbiased estimator for *p* exists with a finite variance. If ℐ is non-singular then the existence of an unbiased estimator of *p* with finite variance is theoretically possible. If Equation (1) holds with equality then the estimator is called *efficient* and the parameter estimate *p̂* is unique.

Consider the set of measurements from *N* sensors *ẑ* ∼ 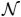
(*z*(*p*), *R_z_*). The Fisher information matrix, in this case, quantifies the amount of information that the observable random vector *ẑ* carries about the unobservable parameter *p*. It is a matrix with the (*i,j*)*^th^* element given by

$${\left(ℐ(p)\right)}_{i,j}=E\left[\frac{\partial }{\partial p_{i}}\ln\;(f_{\hat{z}}(\hat{z};p))\frac{\partial }{\partial p_{j}}\ln\;(f_{\hat{z}}(\hat{z};p))\right]$$

where *p_i_* is the *i^th^* element of the target*'*s location vector *p*. Here, *f_ẑ_*(*ẑ*;*p*) is the likelihood function of *p* given fixed measurements and the natural logarithm of *f_ẑ_*(*ẑ*;*p*) is given by,

$$\ln f_{\hat{z}}(\hat{z};p)=\frac{1}{2}{\left(\hat{z}-z(p)\right)}^{T}R_{z}^{-1}\frac{1}{2}(\hat{z}-z(p))+c$$

where *c* is a constant term independent of *p*. Then the general Fisher information matrix is given by

(2) $$ℐ(p)=\nabla_{p}z{\left(p\right)}^{T}R_{z}^{-1}\nabla_{p}z(p)$$

The analysis of the optimal geometry is subjected to the following constraints:

1. Fixed Uniform Linear Arrays (FULA): One sensor of the linear array is fixed and the distances between consecutive sensors are equal.
2. Uniform Linear Arrays (ULA): The distances between consecutive sensors are equal.
3. Fixed Non-Uniform Linear Arrays (FNULA): One sensor of the linear array is fixed and the distances between consecutive sensors may not be equal.
4. Non-Uniform Linear Arrays (NULA): The distances between consecutive sensors may not be equal.

### 2.1. AoA Based Localization

The measured value of angle (*θ_i_*) is given by,

$${\hat{\theta }}_{i}=\theta_{i}(p)+n_{i}=\arctan(\frac{y_{p}-y_{si}}{x_{p}-x_{si}})+n_{i}$$

where the *arctan* is defined such that *θ_i_*(*p*) ∈ [0, 2π) and the *n_i_* is the measurement error. This error is assumed to be normally distributed with zero mean and variance $\sigma_{\theta }^{2}$, i.e., $n_{i}∼N(0,\sigma_{\theta }^{2})$.

Then using Equation (2), Fisher information matrix (*I_θ_*(*p*)) for *N* number of sensors around the target can be written as,

$${ℐ}_{\theta }(p)=\frac{1}{\sigma_{\theta }^{2}}\overset{N}{\underset{i=1}{\text{∑}}}\frac{1}{r_{i}^{2}}\left[\begin{array}{cc} \sin^{2}\theta_{i} & -\sin\theta_{i}\cos\theta_{i}\\ -\sin\theta_{i}\cos\theta_{i} & \cos^{2}\theta_{i} \end{array}\right]$$

The Fisher information determinant for bearing-only localization can be given as,

(3) $$\text{det}({ℐ}_{\theta }(p))=\frac{1}{\sigma_{\theta }^{4}}\sum_{S}\frac{\sin^{2}(\theta_{j}-\theta_{i})}{r_{j}^{2}r_{i}^{2}}$$

and

$$\text{det}({ℐ}_{\theta }(p))=\frac{1}{4\sigma_{\theta }^{4}}\left[\left(\overset{N}{\underset{i=1}{\text{∑}}}\frac{1}{r_{i}^{2}}\right)-{\left(\overset{N}{\underset{i=1}{\text{∑}}}\frac{\cos2\theta_{i}}{r_{i}^{2}}\right)}^{2}-{\left(\overset{N}{\underset{i=1}{\text{∑}}}\frac{\sin2\theta_{i}}{r_{i}^{2}}\right)}^{2}\right]$$

where *S* = {{*i, j*}} is defined as the set of all combinations of *i* and *j* with *i*, *j* ∈ {1,‥, *N*} and *j*>*i*, implying $\left|S\right|=(\begin{array}{c} N\\ 2 \end{array})$. Here |.| indicates the number of combinations.

### 2.2. Range Based Localization

The measured value of range (*r_i_*) is given by,

$${\hat{r}}_{i}=r_{i}(p)+e_{i}$$

where the *e_i_* is the measurement error and it is assumed to be normally distributed with zero mean and a variance $\sigma_{r}^{2}$ , i.e., $e_{i}∼N(0,\sigma_{r}^{2})$.

Then using Equation (2), the Fisher Information Matrix (*I_r_*(*p*)) for *N* number of sensors around the target can be written as,

$${ℐ}_{r}(p)=\frac{1}{\sigma_{r}^{2}}\overset{N}{\underset{i=1}{\text{∑}}}[\left[\begin{array}{cc} \cos^{2}\theta_{i} & \sin\theta_{i}\cos\theta_{i}\\ \sin\theta_{i}\cos\theta_{i} & \sin^{2}\theta_{i} \end{array}\right]$$

The Fisher information determinant for range-only localization can be given as,

(4) $$\text{det}({ℐ}_{r}(p))=\frac{1}{\sigma_{r}^{4}}\sum_{S}\sin^{2}(\theta_{j}-\theta_{i})$$

and

$$\text{det}({ℐ}_{r}(p))=\frac{1}{4\sigma_{r}^{4}}\left[N^{2}-{\left(\overset{N}{\underset{i=1}{\text{∑}}}\cos2\theta_{i}\right)}^{2}-{\left(\overset{N}{\underset{i=1}{\text{∑}}}\sin2\theta_{i}\right)}^{2}\right]$$

where *S* = {{*i*, *j*}} is defined as the set of all combinations of *i* and *j* with *i*, *j* ∈ {1,‥, *N*} and *j*>*i*, implying $\left|S\right|=(\begin{array}{c} N\\ 2 \end{array})$.

## 3. Optimal Geometries for Inline AoA Sensors

### 3.1. Fixed Uniform Linear Arrays(FULA)

#### Theorem 1

*Consider that a target is at P(x_p_, y_p_) which is b distance away from the x-axis. N number of AoA sensors (one fixed at the origin) are on the x-axis, separated by x distance from each other. The Fisher information determinant for this case is*

(5) $$\text{det}({ℐ}_{x}(p))=\frac{1}{\sigma_{\theta }^{4}}{\overset{N}{\underset{j=2}{\text{∑}}}\overset{j=2}{\underset{i=0}{\text{∑}}}\left\{\frac{b[j-(i+1)]x}{\left[{\left(a-ix\right)}^{2}+b^{2}][{\left(a-[j-1]x\right)}^{2}+b^{2}\right]}\right\}}^{2}$$

*where (a,b) = (x_p_, y_p_)*.

#### Proof 1

*Translating Equation (3) into Cartesian coordinates and rearranging leads to Equation (5)*.

#### Corollary 1

**Figure 2.**
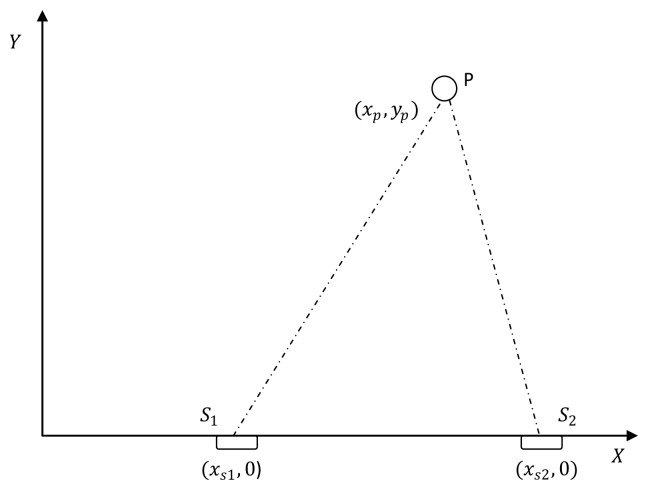
Localization with two sensors (AoA/Range) on x-axis.

*Consider that the target location is P(x_p_, y_p_) and the position of the fixed sensor (S_1_) and the line on which the second sensor to be placed is known (Figure 2). Then the optimal distance between these two sensors is equal to the distance between the fixed sensor and the target (i.e., ‖S_1_ − S_2_‖ = ‖S_1_ − P‖)*.

#### Proof 2

*With no loss of generality consider two sensors, one fixed at the origin (S_1_ = [x_s1_ = 0 0]^T^ ), the other on the x-axis (S_2_ = [x_s2_ 0]^T^ ). The Fisher information determinant for this case is*

(6) $$\text{det}({ℐ}_{x}(p))=\frac{1}{\sigma_{\theta }^{4}}{\left(\frac{-y_{p}x_{s2}}{\left(x_{p}^{2}+y_{p}^{2})[{\left(x_{p}-x_{s2}\right)}^{2}+y_{p}^{2}\right]}\right)}^{2}$$

*By maximizing Equation (6) with respect to x_s2_, it can be shown that, det(ℐ_x_(p)) maximizes when,*

$$x_{s2}=\pm \sqrt{x_{p}^{2}+y_{p}^{2}}$$

*Hence*, ‖*S*_1_− *S*_2_‖ = ‖*S*_1_− *P*‖.

### 3.2. Uniform Linear Arrays (ULA)

#### Theorem 2

*Consider N sensors on a given line b distance away from a target. When x is the distance between consecutive sensors, the optimal localization of the target occurs for the x, which maximize the following Fisher information determinant,*

(7) $$\text{det}({ℐ}_{x}(p))=\frac{1}{\sigma_{r}^{4}}{\overset{N}{\underset{j=2}{\text{∑}}}\overset{j-2}{\underset{i=0}{\text{∑}}}\left\{\frac{\left(b[c-d]x\right)}{\left[{\left(cx\right)}^{2}+b^{2}][{\left(dx\right)}^{2}+b^{2}\right]}\right\}}^{2}$$

*where*

$$c=\frac{N-1}{2}-i$$

*and*

$$d=\frac{N-1}{2}-[j-1]$$

#### Proof 3

*Translating Equation (3) into Cartesian coordinates and rearranging leads to Equation (7)*.

#### Corollary 2

*For two AoA sensors, the optimal sensor separation occurs when* ‖*S*_1_− *S*_2_‖ = ‖ *S*_1_− *P*‖ = ‖*S*_2_−* P*‖.

#### Proof 4

*Using Equation (3), the Fisher information determinant for this case is*

(8) $$\text{det}({ℐ}_{x}(p))={\left\{\frac{y_{p}(x_{s2}-x_{s1})}{\left[{\left(x_{p}-x_{s1}\right)}^{2}+x_{p}^{2}][{\left(x_{p}-x_{s2}\right)}^{2}+x_{p}^{2}\right]}\right\}}^{2}$$

*It can be shown that the maximum of Equation (8) occurs when*

$$x_{s1}=x_{p}-y_{p}/\sqrt{3}$$

*and*

$$x_{s2}=x_{p}+y_{p}/\sqrt{3}$$

*when this relationship holds for the optimal sensor separation,* ‖*S*_1_ − *S*_2_‖ = ‖*S*_1_ − *P*‖ = ‖*S*_2_ −*P*‖.

### 3.3. Fixed Non-Uniform Linear Arrays (FNULA)

Suppose a target (*P*) is to be localized using *N* number of linear sensors (*S*_1_, *S*_2_, ,… , *S_N_*). Translating Equation (3) in to Cartesian coordinates, it can be shown that the Fisher information determinant for this case is

$$\text{det}({ℐ}_{\theta }(p))=\frac{1}{\sigma_{\theta }^{4}}{\sum_{S}\left\{\frac{b\left\|S_{j}-S_{i}\right\|}{{\left\|S_{j}-P\right\|}^{2}{\left\|S_{i}-P\right\|}^{2}}\right\}}^{2}$$

where *b* is the distance between the target and the linear array and *S* = {{*i*, *j*}} is defined as the set of all combinations of *i* and *j* with *i, j* ∈ {1,‥, *N*} and *j*>*i*, implying $\left|S|=(\begin{array}{c} N\\ 2 \end{array}\right)$.

Finding the optimal sensor separations becomes an (*N* − 1)-dimensional optimization problem. Finding the solutions is mathematically challenging when *n*>*3* and the solutions for the *n* = 3 case have been found in [40], which is a two-dimensional optimization problem.

#### 3.3.1. Non-Uniform Linear Arrays (NULA)

##### Theorem 3

*Consider N number of AoA sensors on a given line b distance away from a target. At the optimal geometry, sensors form an equilateral triangle with the target*.

1. *N is even; N/2 sensors overlap at each corner of the triangle located on the line*.
2. *N is odd; (N − 1)/2 and (N + 1)/2 sensors overlap at each corner of the triangle located on the line respectively*.

##### Proof 5

**Figure 3.**
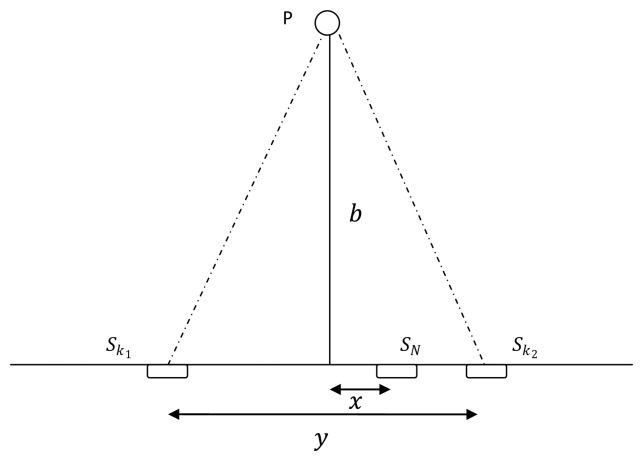
Localization with *N* number of AoA sensors.

*Consider the sensor-target geometry shown in Figure 3. When the total number of sensors used for localization is odd (N ∈ {3,5, 7,…}); assume that (N − 1)/2 number of sensors are overlapping at each corner of the triangle (S_k1_ and S_k2_), which are y distance apart and the remaining sensor is x distance away from the symmetric axis. Using Equation (3) the Fisher information determinant for this case can be written as*

(9) $$\begin{array}{ll} \text{det}({ℐ}_{x,y}(p)) & =\left(\frac{N-1}{2}\right)\frac{b^{2}{\left(y+x\right)}^{2}}{{\left[(y^{2}+b^{2})(x^{2}+b^{2})\right]}^{2}}\\ & +\left(\frac{N-1}{2}\right)\frac{b^{2}{\left(y-x\right)}^{2}}{{\left[(y^{2}+b^{2})(x^{2}+b^{2})\right]}^{2}}\\ & +{\left(\frac{N-1}{2}\right)}^{2}\frac{b^{2}{\left(2y\right)}^{2}}{{\left(y^{2}+b^{2}\right)}^{4}} \end{array}$$

*It can be shown that Equation (9) is at maximum when* $x=b/\sqrt{3}$ and $y=b/\sqrt{3}\forall N\in \{3,5,7,\ldots \}$. *When the total number of sensors used for localization is even (N ∈ {2,4,6,…}); assume that N/2 and N/2 − 1 number of sensors are overlapping at each corner of the triangle (S_k1_ and S_k2_), which are y distance apart and the remaining sensor is x distance away from the symmetric axis. Using Equation (3) the Fisher information determinant for this case can be written as*

(10) $$\begin{array}{ll} \text{det}({ℐ}_{x,y}(p)) & =\left(\frac{N}{2}-1\right)\frac{b^{2}{\left(y+x\right)}^{2}}{[(y^{2}+b^{2}){\left(x^{2}+b^{2}\right]}^{2}}\\ & +\left(\frac{N}{2}\right)\frac{b^{2}{\left(y-x\right)}^{2}}{[(y^{2}+b^{2}){\left(x^{2}+b^{2}\right]}^{2}}\\ & +\frac{N}{2}\left(\frac{N}{2}-1\right)\frac{b^{2}{\left(2y\right)}^{2}}{{\left(y^{2}+b^{2}\right)}^{4}} \end{array}$$

*It can be shown that Equation (10) reaches its maximum when* $x=b/\sqrt{3}$ *and* $y=b/\sqrt{3}\forall N\in \{2,4,6,\ldots \}$. *Then it is clear that for any N ≥ 2,* $x=b/\sqrt{3}$ *and* $y=b/\sqrt{3}$ *provide the optimal geometry for AoA-based localization, which is an equilateral triangle*.

## 4. Optimal Geometries for Inline Range Sensors

### 4.1. Fixed Uniform Linear Arrays (FULA)

#### Theorem 4

*Consider that a target is at P(x_p_, y_p_) and b distance away from the x-axis. N number of linear range sensors (one fixed at the origin), separated by x distance from each other, are on the x-axis. The Fisher information determinant for this case is*

(11) $$\det({ℐ}_{x}(p))=\frac{1}{\sigma_{r}^{4}}\overset{N}{\underset{j=2}{\text{∑}}}\overset{j-2}{\underset{i=0}{\text{∑}}}\left\{\frac{{\left(b[j-(i+1)]x\right)}^{2}}{\left[{\left(a-ix\right)}^{2}+b^{2}][{\left(a-[j-1]x\right)}^{2}+b^{2}\right]}\right\}$$

*where* (*a*, *b*) = (*x_p_*, *y_p_*).

#### Proof 6

*Translating (4) into Cartesian coordinates and rearranging leads to (11)*.

#### Corollary 3

*Consider that the target is at P*(*x_p_, y_p_*) *and the position of one sensor (fixed) and the line on which the second sensor to be placed is known. The optimal geometry occurs when the angle subtended by the sensors at the target is π/2 (i.e., S_1_P̂S_2_ = π/2)*.

#### Proof 7

*With no loss of generality consider two sensors, one fixed at the origin (S*_1_ = [*x_s1_* = 0 0]*^T^*) *and the other on the x-axis (S*_2_ = [*x_s_*_2_ 0]*^T^*). *The target is at P*(*x_p_, y_p_*) *as shown in Figure 2. The Fisher information determinant for this case is*

(12) $$\text{det}({ℐ}_{x}(p))=\frac{1}{\sigma_{r}^{4}}\left(\frac{{\left(-y_{p}x_{s2}\right)}^{2}}{\left(x_{p}^{2}+y_{p}^{2})[{\left(x_{p}-x_{s2}\right)}^{2}+y_{p}^{2}\right]}\right)$$

*By maximizing the (12) with respect to x_s__2_, it can be shown that, det(ℐ_x_(p)) maximizes when*,

$$x_{s2}=\pm \frac{x_{p}^{2}+y_{p}^{2}}{x_{p}}.$$

*This proves that the optimal geometry occurs when the angle subtended by the sensors at the target is π/2*.

### 4.2. Uniform Linear Arrays (ULA)

#### Theorem 5

*Consider N sensors on a given line that are b distance away from the target. With equal distance x between consecutive sensors, the optimal localization of the target occurs for the x, which maximizes the following Fisher information determinant*,

(13) $$\text{det}({ℐ}_{x}(p))=\frac{1}{\sigma_{r}^{4}}\overset{N}{\underset{j=2}{\text{∑}}}\overset{j-2}{\underset{i=0}{\text{∑}}}\left\{\frac{{\left(b[c-d]x\right)}^{2}}{\left[{\left(cx\right)}^{2}+b^{2}][{\left(dx\right)}^{2}+b^{2}\right]}\right\}$$

*where*

$$c=\frac{N-1}{2}-i$$

*and*

$$d=\frac{N-1}{2}-[j-1]$$

#### Proof 8

*Translating Equation (4) into Cartesian coordinates and rearranging leads to Equation (13)*.

#### Corollary 4

*For two range sensors, the optimal sensor target geometry occurs when the angle subtended by the sensors at the target is* π/2 (i.e., *S*_1_*P̂S*_2_ = π/2).

#### Proof 9

*Using Equation (4), the Fisher information determinant for this case is*

(14) $$\text{det}({ℐ}_{x}(p))=\frac{{\left[y_{p}(x_{s2}-x_{s1})\right]}^{2}}{\left[{\left(x_{p}-x_{s1}\right)}^{2}+x_{p}^{2}][{\left(x_{p}-x_{s2}\right)}^{2}+x_{p}^{2}\right]}$$

*It can be shown that the maximum of (14) occurs when*

$$x_{s2}=\frac{x_{p}^{2}+y_{p}^{2}-x_{p}x_{s1}}{x_{p}-x_{s1}}$$

*when this relationship holds for the optimal sensor target geometry, the angle subtended by the sensors at the target is* π/2.

This result agrees with the geometrical relationships obtained in [11], where they prove that, for two range sensors, the optimal sensor-target geometry is unique and occurs when the angle subtended by the sensors at the target is π/2.

### 4.3. Fixed Non-Uniform Linear Arrays (FNULA)

Suppose a target (*P*) is to be localized using *N* number of linear sensors (*S*_1_, *S*_2_,…, *S_N_*). Translating Equation (4) into Cartesian coordinates, it can be shown that the Fisher information determinant for this case is

$$\text{det}({ℐ}_{r}(p))=\frac{1}{\sigma_{r}^{4}}{\sum_{S}\left\{\frac{b\left\|S_{j}-S_{i}\right\|}{\left\|S_{j}-P\right\|\left\|S_{i}-P\right\|}\right\}}^{2}$$

where *b* is the distance between the target and the linear array whilst *S* = {{*i*, *j*}} is defined as the set of all combinations of *i* and *j* with *i*, *j* ∈ {1, ‥ , *N*} and *j*>*i*, implying $\left|S\right|=(\begin{array}{c} N\\ 2 \end{array})$.

Finding the optimal sensor separation becomes an (*N* − 1)-dimensional optimization problem.

### 4.4. Non-Uniform Linear Arrays (NULA)

Suppose a target (*P*) is to be localized using *N* number of inline sensors (*S*_1_, *S*_2_,…, *S_N_*). Translating Equation (4) into Cartesian coordinates, it can be shown that the Fisher information determinant for this case is

$$\text{det}({ℐ}_{r}(p))=\frac{1}{\sigma_{r}^{4}}{\sum_{S}\left\{\frac{b\left\|S_{j}-S_{i}\right\|}{\left\|S_{j}-P\right\|\left\|S_{i}-P\right\|}\right\}}^{2}$$

where *b* is the distance between the target and the linear array whilst *S* = {{*i*, *j*}} is defined as the set of all combinations of *i* and *j* with *i*, *j* ∈ {1, ‥ , *N*} and *j*>*i*, implying $\left|S\right|=(\begin{array}{c} N\\ 2 \end{array})$.

Finding the optimal sensor separation becomes an *N*-dimensional optimization problem, which will be discussed in detail in our future research.

## 5. Simulations

### 5.1. Simulations Related to Theorem 1

**Figure 4.**
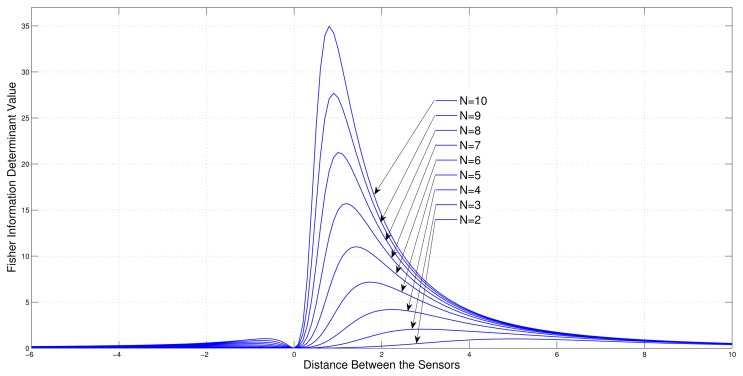
Variation of Fisher information determinant value with the distance between two adjacent sensors of ULA for different number of AoA sensors (One sensor fixed).

Consider a sensor-target geometry where one sensor (*S*_1_) is fixed at the origin and the other sensors (*S*_2_,*S*_3_, … *S_N_*) are free to be located on the *x*-axis with equal distance from each other. The target is at *P* = [3 4]*^T^*. Figure 4 shows the variation of the Fisher information determinant value with the distance between the sensors for different numbers of sensors.

It can be seen from the figure that when the number of sensors is increased, the Fisher information determinant value increases and the inter-sensor distance decreases for optimal localization, which is unique for a given number of sensors.

### 5.2. Simulations Related to Theorem 2

#### 5.2.1. Two Adjustable Sensors

**Figure 5.**
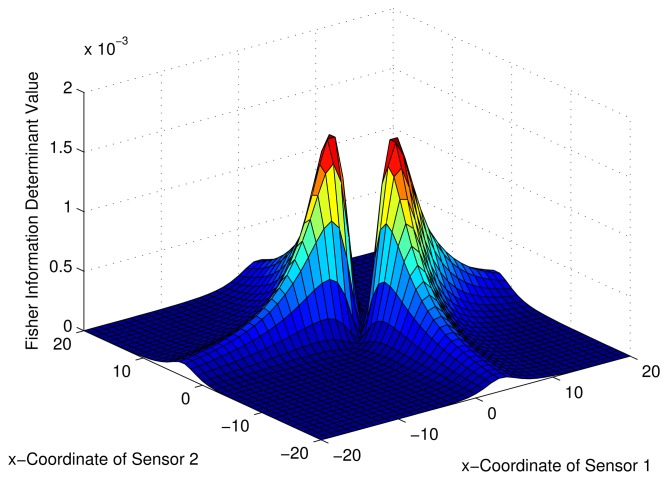
Variation of Fisher information determinant value with the AoA sensors positions.

**Figure 6.**
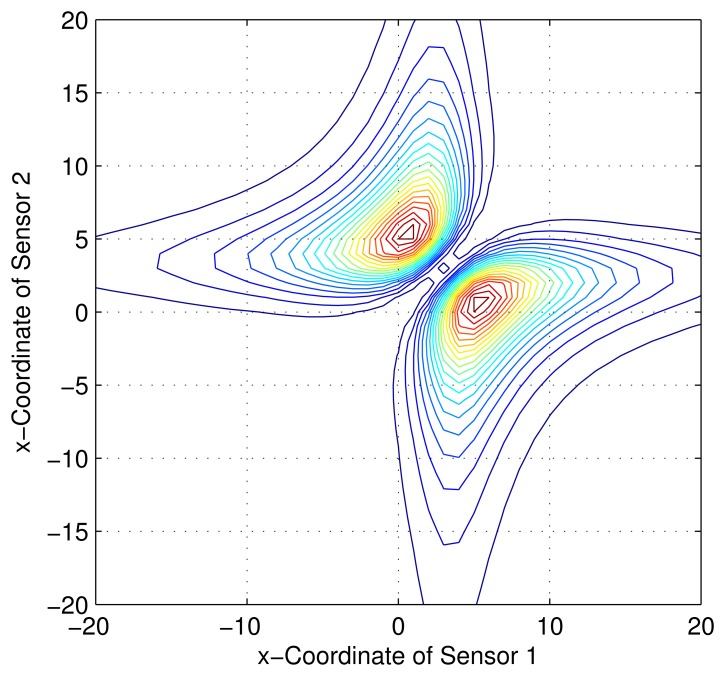
Variation of Fisher information determinant value with the AoA sensors positions (Contour plot).

Consider a sensor-target geometry as depicted in the Figure 2, where the sensors (*S*_1_) and (*S*_2_) are located anywhere on the *x*-axis. The target is at *P* = [3 4]*^T^*. The variation of the Fisher information determinant value with the positions of the two sensors is depicted in Figure 5 and the corresponding contour plot in Figure 6. It can be seen that the Fisher information value is maximized when $x_{s1}=\frac{9-4\sqrt{3}}{3}$ and $x_{s2}=\frac{9+4\sqrt{3}}{3}$ (*Corollary 2*). When *x_s_*_1_ and *x_s_*_2_ attain these values, the geometry of the sensor-target configuration is an equilateral triangle. (*i.e.*, ‖*S*_1_ − *S*_2_‖ = ‖*S*_1_ − *P*‖ = ‖*S*_2_ − *P*‖).

#### 5.2.2. ULA with Multiple Adjustable Sensors

**Figure 7.**
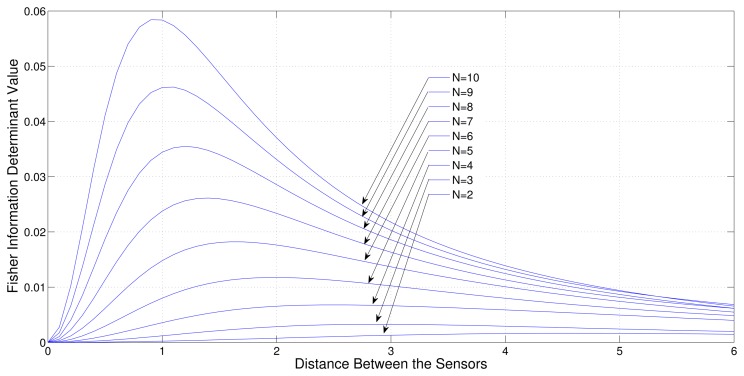
Variation of Fisher information determinant value with the distance between two adjacent sensors of ULA for different number of AoA sensors (All adjustable).

Consider a sensor target geometry as depicted in the Figure 3, where all the sensors are equally separated by *x* distance and the distance to the target from the line on which the sensors are placed is 4. The variation of Fisher information determinant value with respect to *x* is depicted in Figure 7 for different numbers of sensors.

It can be seen from the figure that when the number of sensors is increased, the Fisher Information determinant value increases and the distance between the sensors decreases for optimal localization, which is unique for a given number of sensors.

### 5.3. Simulations Related to Theorem 4

**Figure 8.**
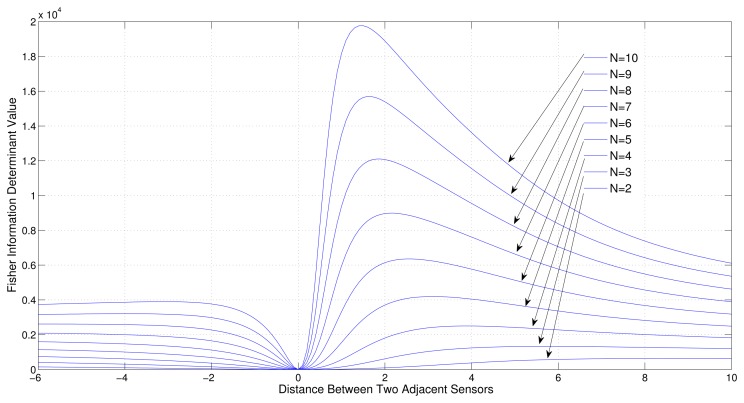
Variation of Fisher information determinant value with the distance between two adjacent sensors of ULA for different number of range sensors (One sensor fixed).

Consider a sensor-target geometry where one sensor (*S*_1_) is fixed at the origin and the other sensors (*S*_2_,*S*_3_, … *S_N_*) are located anywhere on the *x*-axis keeping the same distance from each other. The target is at *P* = [3 4]*^T^*. Figure 8 shows the variation of Fisher information determinant value with the distance between the sensors for different number of sensors.

It can be seen from the figure that when the number of sensors is increased, the Fisher information determinant value increases and the distance between the sensors decreases for optimal localization, which is unique for a given number of sensors.

### 5.4. Simulations Related to Theorem 5

#### 5.4.1. Two Adjustable Sensors

**Figure 9.**
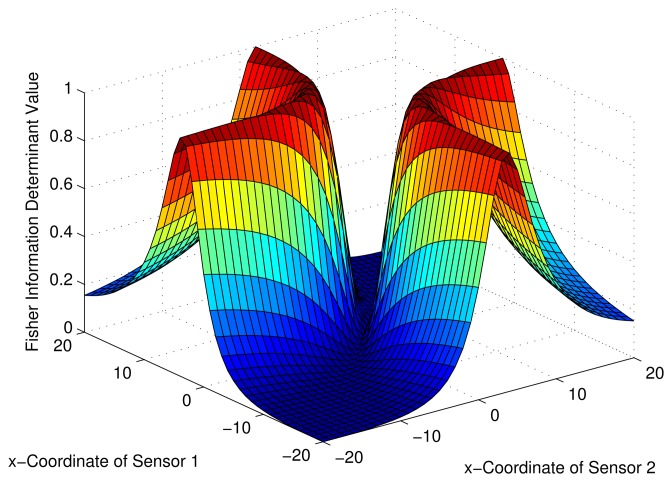
Variation of Fisher information determinant value with the range sensors positions.

**Figure 10.**
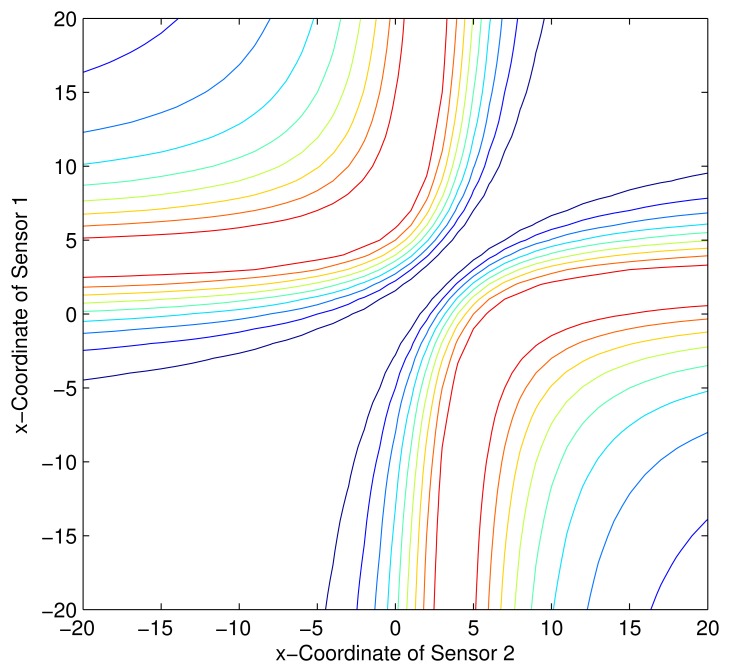
Variation of Fisher information determinant value with the range sensors positions (Contour plot).

Consider a sensor-target geometry as depicted in the Figure 2, where the sensors (*S*_1_) and (*S*_2_) are located anywhere on the *x*-axis. The target is at *P* = [3 4]*^T^*. The variation of the Fisher information determinant value with the locations of the two sensors is depicted in Figure 9 and the corresponding contour plot in Figure 10. It can be seen that the Fisher information value maximizes when *x_s_*_1_ and *x_s_*_2_ satisfy (14)(*Corollary 4*).

#### 5.4.2. ULA with Multiple Adjustable Sensors

**Figure 11.**
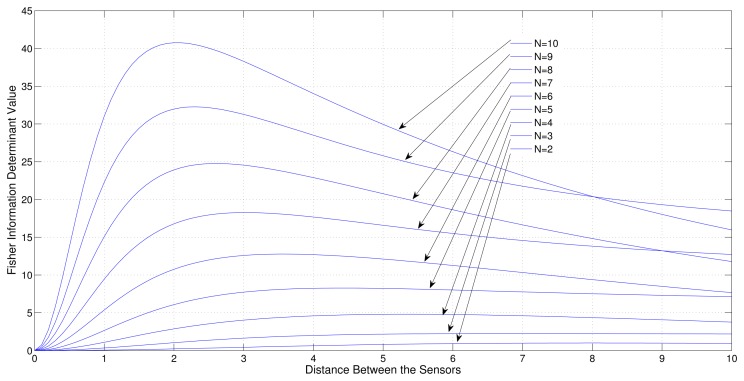
Variation of Fisher information determinant value with the distance between two adjacent sensors of ULA for different number of range sensors (All adjustable).

Consider a sensor-target geometry where all the sensors are equally separated by *x* distance and the distance to the target from the line on which the sensors are placed is 4. Variation of the Fisher Information determinant value with respect to *x* is depicted in Figure 11 for different number of sensors.

It can be seen from the simulation that when the number of sensors increases, the Fisher information determinant value increases and the distance between the sensors decreases for optimal localization, which is unique for a given number of sensors.

## 6. Conclusions

In this paper, we have provided a characterization of optimal sensor-target geometry for linear arrays of AoA and range sensors in passive localization problems in ℝ^2^. We have mainly discussed two generic problems of fully adjustable linear sensor arrays and the case of an array, where the sensors are free to be moved with respect to a fixed sensor. Cramer–Rao lower bound and the corresponding Fisher information matrices are used to analyze the sensor target geometry for optimal localization.

The perfect knowledge of the emitter position should be available in the theoretical development for determining optimal sensor placement. Even though in practical applications this information is not available, a rough estimate of the likely region of the emitter is sufficient in determining the sensor positions to obtain improved localization results. Hence the results of this paper can be utilized to establish guidelines for linear sensor placement leading to improved performance.

The analysis given in this paper is also related to optimal path planning and trajectory control of mobile sensors for localization, e.g., see [15,20,25].
